# 

**Published:** 1908-06

**Authors:** 


*The following donations have been received since the publication of the List in March:*

*May* 31*st*, 1908.

Professor Walker Hall (1) .. .. .. .. 1 volume.

J. Taylor .. .. .. .. Unbound periodicals.

### SIXTY-EIGHTH LIST OF BOOKS.

The titles of books mentioned in previous lists are not repeated.

The figures in brackets refer to the figures after the names of the donors and show by whom the volumes were presented. The books to which no such figures are attached have either been bought from the Library Fund, or received through the *Journal*.

**Arthur, W. B.** .. *Supplementary First Aid to Miners* .. .. 1908

**Atkinson, S. B.** .. *The Law in General Practice* .. .. .. 1908

**Bardswell and J. E. Chapman, N. D.** *Diets in Tuberculosis* .. .. 1908

**Brunner, C.** .. .. *Tuberculose, Aktinomykose, Syphilis des Magendarmkanals* .. .. .. .. .. .. 1907

**Campbell, J.** .. .. *A Text-Book of Treatment* .. Part I. 1908

**Chapman, N. D. Bardswell and J. E.** *Diets in Tuberculosis* .. .. 1908

**Edmunds, A.** .. .. *Glandular Enlargement* .. .. .. .. .. .. 1908

*Encyclopedia and Dictionary of Medicine and Surgery, Green's* Vol. VII. [1908]

**Freyer, P. J.** .. .. *The Surgical Diseases of the Urinary Organs* 1908

**Harman, N. B.** .. *Aids to Ophthalmology* .. .. .. 4th Ed. 1908

**Howell, H. W. Wilson and C. M. H.** *Movable Kidney* .. .. 1908

**Jones, J. A.** .. .. *A Short Practice of Aural Surgery* .. .. .. 1908

**Keith, S. and G. E.** *Cancer; Relief of Pain and Possible Cure* .. 1908

**Kenealy, A.** .. .. *The Failure of Vivisection and the Future of Medical Research* .. .. .. .. .. .. [1908]

**Kenwood, H. R.** .. *Public Health Laboratory Work* .. 4th Ed. 1908

**Leedham-Green, C.** *The Treatment of Gonorrhœa in the Male* 2nd Ed. 1908

**Lloyd, W.** .. .. *Hay-Fever, Hay-Asthma* .. 2nd Ed. 1908

**Maissoneuve, P.** .. *The Experimental Prophylaxis of Syphilis* (Tr. by F. L. de Verteuil) .. .. .. 1908

**Marshall, C. F.** .. *Golden Rules of Venereal Disease* .. .. [1908]

**May and C. Worth, C. H.** *Diseases of the Eye* .. 2nd Ed. 1908

**Murphy, D'A. Power and J. K.** [Eds.] *A System of Syphilis* Vol. I. 1908

**Murray, R. W.** .. *Hernia* .. .. .. .. .. .. .. .. .. 1908

**Power and J. K. Murphy, D'A.** [Eds.] *A System of Syphilis* Vol. I. 1908

**Quain, [ ]** .. *Elements of Anatomy.* 11th Ed. Vol. I. By T. H. Bryce .. .. .. .. .. .. 1908

**Russell, W.** .. .. *Arterial Hypertonus, Sclerosis and Blood-Pressure* .. .. .. .. .. .. .. .. .. 1907

**Sawyer, J. E. H.** .. *Physical Signs* .. .. .. .. .. .. .. 1908

**Sherran, J.** .. .. *Injuries of Nerves and their Treatment* .. .. 1908

**Thomson, H. C.** .. *Diseases of the Nervous System* .. .. .. 1908

**Vincent, R.** .. .. *Lectures on Babies* .. .. .. .. .. .. .. 1908

**Walker, N.** .. .. *An Introduction to Dermatology* .. 4th Ed. 1908

**Wallace, C. S.** .. *Prostatic Enlargement* .. .. .. .. .. .. 1907

**Williams, C.** .. .. *Insanity: its Causes and Prevention* .. .. 1908

**Williams, L.** .. .. *Minor Maladies and their Treatment* 2nd Ed. 1908

**Williamson, R. T.** *Diseases of the Spinal Cord* .. .. .. .. .. 1908

**Wilson and C. M. H. Howell, H. W.** *Movable Kidney* .. .. .. 1908

**Worth, C. H. May and C.** *Diseases of the Eye* .. .. .. 2nd Ed. 1908

**Wrench, G. T.** .. *Rotunda Practice of Midwifery* .. .. .. 1908

**Young, J.** .. .. *A Chronology of Medical History* .. .. [N.D.]

### TRANSACTIONS, REPORTS, JOURNALS, &c.

| | | |
|---|---|---|
| Albany Medical Annals | Vol. XXVII. | 1906 |
| American Journal of Obstetrics, The | Vol. LVI. | 1907 |
| American Journal of Ophthalmology, The | Vol. XXIV. | 1907 |
| American Journal of the Medical Sciences, The | Vol. CXXXIV. | 1907 |
| American Medicine | N.S., Vol. II. | 1907 |
| Annales de Dermatologie et de Syphiligraphie | Tom. VIII. | 1907 |
| Archives of Pediatrics | Vol. XXIV. | 1907 |
| Australasian Medical Gazette, The | Vol. XXVI. | 1907 |
| Bookseller, The | | 1907 |
| Bristol Medico-Chirurgical Journal, The | Vol. XXV. | 1907 |
| British Journal of Children's Diseases, The | Vol. IV. | 1907 |
| British Journal of Dermatology, The | Vol. XIX. | 1907 |
| Bulletin de l'Académie royale de Médecine de Belgique | Tom. XXI. | 1907 |
| Bulletin de la Société française de Dermatologie | | 1907 |
| Catalogue of Books for 1907, The English | | 1908 |
| College of Physicians of Philadelphia, Transactions of the | Vol. XXIX. | 1907 |
| Gazette des Hôpitaux | | 1907 |
| Harvard Medical School, Contributions from the Department of Neurology | Vol. III. | 1908 |
| Hospitals— | | |
| Bristol Royal Infirmary, Second Annual Pathological Report (1) | | 1907 |
| St. Bartholomew's Hospital Reports | Vol. XLIII. | 1908 |
| Indian Medical Gazette, The | Vol. XLII. | 1907 |
| Journal of Hygiene, The | Vol. VII. | 1907 |
| Journal of Laryngology, The | Vol. XXII. | 1907 |
| Journal of Medical Research, The | Vol. XVII. | 1907–08 |
| Journal of Mental Science. The | Vol. LIII. | 1907 |
| Journal of Nervous and Mental Disease, The | Vol. XXXIV. | 1907 |
| Journal of Obstetrics and Gynæcology, The | Vol. XII. | 1907 |
| Journal of the American Medical Association, The | Vol. XLIX. | 1907 |
| Journal of the Sanitary Institute | Vol. XXVIII. | 1907 |
| Journal of Tropical Medicine, The | Vol. X. | 1907 |
| Library Association Record, The | Vol. IX. | 1907 |
| Luzerne County Medical Society, Transactions of the | Vol. XV. | 1907 |
| Medical Annual, The | | 1908 |
| Medical Chronicle, The | N.S., Vols. XIII., XIV. | 1907–08 |
| Medical Society's Transactions, The | Vol. XXX. | 1907 |
| Montreal Medical Journal, The | Vol. XXXVI. | [1907] |
| Nord médical, Le | | 1907 |
| Obstetrical Transactions | Vol. XLIX. | 1908 |
| Post-Graduate, The | Vol. XXII. | 1907 |
| Progrès médical, Le | | 1907 |

| | | |
|---|---|---|
| Progressive Medicine .. .. .. .. .. .. .. .. | Vol. I. | 1908 |
| Public Health .. .. .. .. .. .. .. | Vols. XIX., XX. | 1906–08 |
| Revue génerale d'Ophtalmologie .. .. .. .. | Tom. XXVI. | 1907 |
| Society for the Study of Disease in Children, Reports of the | Vol. VII. | 1906–07 |
| Therapeutic Gazette, The .. .. .. .. .. .. .. .. | Vol. XXXI. | 1907 |
| Wiener klinische Wochenschrift.. .. .. .. .. .. .. .. .. .. | | 1907 |

### MEETINGS OF SOCIETIES.

### Bristol Medico-Chirurgical Society.

### Local Medical Notes.

**University College, Bristol.**—Recent examination successes:

M.B., B.S. Lond.—C. E. K. Herapath, F. S. Scott, L. J. Short, A. N. Thomas, and R. E. Thomas.

F.R.C.S. Eng.—A. Rendle Short, M.B., B.S. Lond.

M.R.C.P. Lond.—Carey F. Coombs, M.D. Lond.

Intermediate M.B., B.S. Lond.—R. M. Hiley. *Organic Chemistry:* W. Salisbury. *Inorganic Chemistry:* E. S. Abraham.

Conjoint Board.—*Practical Pharmacy:* M. W. Morrison. *Biology:* C. Kingston. *Anatomy and Physiology:* C. H. Hart. *Medicine:* E. R. Sircom, E. J. Dermott,* P. S. Tomlinson,* E. V. Connellan,* F. S. Scott.* *Midwifery:* E. Christofferson, F. C. Nicholls, E. E. Davies, B. Stone, C. Clarke.

L.D.S.—*Chemistry and Physics:* R. A. Slade.

**Bristol Dental Students' Society.**—A society with this title has been formed amongst the students for the reading and discussion of papers on dentistry and allied subjects. The meetings are held monthly, and all present and past students of the school are eligible for membership. The subscription is only a nominal one, as it is hoped to obtain the support of all the past and present students. Mr. Ackland has been elected president for this session.

**Anatomical and Anthropological Society.**—With Professor Fawcett as President, and many eminent professors as Vice-Presidents, this Society, comprising the majority of the students of the Faculty of Medicine, has held several successful meetings during the past winter session. The papers read by the students have reached a high degree of excellence, many of them being illustrated by lantern slides. During the present summer session the meetings will be resumed on Fridays at 5 p.m.

**Bristol Royal Infirmary.**—F. H. Rudge, M.R.C.S., L.R.C.P., has been appointed House Physician.

**Bristol General Hospital.**—The following appointments have recently been made:—*Senior House Surgeon:* E. Maynard, F.R.C.S., has been appointed. *House Physician:* J. W. J. Willcox, M.B., B.S. Lond. *House Surgeon:* A. E. Iles, M.R.C.S., L.R.C.P. *Assistant House Physician:* L. J. Short. *Casualty Officer:* P. S. Tomlinson, M.R.C.S., L.R.C.P.

**Bristol Royal Hospital for Children and Women.**—The annual meeting of the subscribers to this institution was held on

* Denotes qualification.

March 31st, under the Presidency of Lord Winterstoke. The medical report stated that during 1907 the in-patients numbered 785 (727 children and 58 women), a decrease compared with 1906. The out-patients numbered 4,662, as compared with 5,500 in the previous year. The financial statement showed that the total adverse balance against the institution was now about £4,000. The committee states that as a result of its special appeal for £10,000 (which will be utilised for freeing the hospital from debt, for building a small scarlet fever annexe, and for improvements in the out-patient department), the sum of £5,209 has already been received.

**Bristol Queen Victoria Jubilee Convalescent Home.**—The eighth annual report of this institution has just been issued, and shows the usefulness of the institution. The number of patients admitted in 1907 was 1,533, making a total since the foundation of 11,163. The average stay of each patient was seventeen and a half days, and the average increase in weight during their stay was just under three pounds. With regard to the finances, a balance of £472 was carried to the current year. The income amounted to £3,158, and the expenditure to £2,641. A sum of £400 has been placed to the special repair fund. The medical officer reports that of the 1,533 patients admitted, 1,447 were discharged much better for their stay, 58 were unimproved in health, whilst 28 were returned to the institution from which they were sent for further treatment. There was no loss by death during the year.

**Medical Inspection of School Children.**—At the meeting of the Bristol Education Committee, held on April 30th, it was decided to appoint five "part-time" medical officers as inspectors of school children at salaries of £100 per annum, one at least of these officers to be a woman. It is proposed that each officer shall devote to this special work between two and three hours per day for three days of the week. In addition two health visitors, who will be certificated nurses, are to be appointed, at annual salaries of £70 each.

**Gloucester Infirmary.**—The annual meeting of the subscribers to the Gloucester Infirmary and Gloucester Eye Institution was held on March 26th. The medical report stated that 1,390 patients were admitted during 1907, an increase of 91 as compared with 1906. The out-patients numbered 8,412, being 500 less than in the previous year. The average cost per bed was £84. The financial statement showed that the deficit of £956 remaining at the close of the year 1906 was now reduced to £186.

**University College Public Health Laboratory.**—For the inaugural meeting of this laboratory, the Council were fortunate in obtaining the assistance of Lt.-Col. R. H. Firth, R.A.M.C., and Professor Dreyer, of Oxford.

The large attendance of medical officers of health, and of those interested in clinical pathology, evidenced the interest taken in the project. Dr. Michell Clarke presided, and explained that the Council of the College had taken over the clinical research work recently carried on by Professor Stanley Kent, and had appointed Dr. J. Fortescue-Brickdale as Director. A Consulting Board of Experts had also been constituted, in order that the field of investigations might be widened. He then introduced Lt.-Col. R. H. Firth, who gave a forcible and telling address upon the " Sanitary Service of the Territorial Army." Later, Professor Dreyer described his methods for the performance of Widal reactions in typhoid fever.

**University of Bristol.**—A further step towards the granting of a Charter for the University of Bristol was made when the City Council, at their last meeting, pledged themselves to support financially the scheme.

In moving the adoption of the recommendation of the Committee appointed to consider the question of giving financial support to the proposed University, Dr. Cook, in his illuminating speech, left no part of the case for a University in Bristol untouched. He laid stress on the tardy awakening of English public opinion to the value of the fullest scientific and technical instruction. We in England have allowed Germany a long lead, but we are rapidly overtaking our deficiencies, and perhaps our very delays have enabled us to avoid some of Germany's mistakes. The old Universities have within recent years made great advances in equipment, and they contemplate greater. The new Universities—to which Bristol is soon to add one more—have entered on a field peculiarly their own, with every prospect of doing brilliant and fruitful work in it. To make the educational system of our ancient city complete and self-sustained, from the bottom to the top, ought to be an object of pride and ambition to every citizen. It cannot be so while students have to go elsewhere for degrees. And there is an " atmosphere " favourable to culture which comes from the establishment of a local University, as the industrial cities of the North have proved. The promise of civic support is a condition precedent to the granting of a University Charter. We are glad that the promise has been given, although the extent of the aid is left for future decision. The city has a great opportunity, and is seizing it. There were few evidences at the meeting of the " mistrust " which the Bishop of Hereford deprecated in his recent eloquent appeal. We hope they will not obtrude themselves later. Heavily rated though Bristol is, the prospect of a halfpenny, or even a penny, more by and by for so great a purpose is little compared with the prospective gain. " From the lowest point of material progress," as Mr. G. E. Davies very well put it, " the establishment of a University will be of enormous advantage."

### *PUBLICATIONS RECEIVED.*

From THE AMBROSE CO. LIMITED, London:
*Insanity: its Causes and Prevention.* By CHARLES WILLIAMS. 1908. Price, 2s. 6d. net.

From SIDNEY APPLETON, London:
*A Text-book of Treatment.* Part I.—*Obstetrics and Gynecology.* By JOHN CAMPBELL, M.A., M.D. 1908. Price, 10s. net.

From EDWARD ARNOLD, London:
*Movable Kidney.* By HAROLD W. WILSON, M.B., B.S., and C. M. HINDS HOWELL, M.A., M.B. 1908. Price, 4s. 6d. net.

From BAILLIÈRE, TINDALL & COX, London:
*Minor Maladies and their Treatment.* By LEONARD WILLIAMS, M.D. Second Edition, revised and enlarged. 1908. Price, 5s. net.
*Aids to Ophthalmology.* By N. BISHOP HARMAN, M.A., M.B. Fourth Edition. 1908. Price, 2s. 6d. net.
*Clinical Lectures on the Surgical Diseases of the Urinary Organs.* By P. J. FREYER, M.A., M.D., M.Ch. 1908. Price, 12s. 6d. net.
*Lectures on Babies.* By RALPH VINCENT, M.D., B.S. 1908. Price, 2s. 6d. net.
*The Treatment of Gonorrhœa in the Male.* By CHARLES LEEDHAM-GREEN, M.B. Second Edition. 1908. Price, 5s. net.
*Physical Signs of Diseases of the Thorax and Abdomen.* By JAMES E. H. SAWYER, M A., M.D. 1908.
*A Manual of Diseases of the Eye.* By CHARLES H. MAY, M.D., and CLAUD WORTH. Second Edition. 1908. Price, 10s. 6d. net.
*Diphtheria in Practice.* By W. F. LITCHFIELD. 1908. Price, 3s. 6d. net.

From ERNEST BELL, London:
*The Failure of Vivisection and the Future of Medical Research.* By ARABELLA KENEALY.

From "THE BIBLIOPHILE" OFFICE, London:
*The Bibliophile.* Vol. I. No. 1. March, 1908.

From ADAM AND CHARLES BLACK, London:
*Cancer: Relief of Pain and Possible Cure.* By SKENE KEITH, M.B., and GEORGE E. KEITH, M.B., C.M. 1908. Price, 2s. 6d. net.

From THE CAMBRIDGE UNIVERSITY PRESS, Cambridge:
*Parasitology.* Vol. I. No. 1. March, 1908.

From CASSELL & COMPANY, LIMITED, London:
*Diseases of the Nervous System.* By H. CAMPBELL THOMSON, M.D. 1908. Price, 10s. 6d.

From J. & A. CHURCHILL, London:
*Hernia: its Cause and Treatment.* By R. W. MURRAY. 1908. Price, 4s. 6d. net.

From EDWARD EVERARD, Bristol:
*A Chronology of Medical History.* By JAMES YOUNG, M.D. [N.D.]

From HENRY FROWDE AND HODDER AND STROUGHTON, London:
*Rotunda Midwifery for Nurses and Midwives.* By G. T. WRENCH, M.D. 1908. Price, 6s. net.
*Diets in Tuberculosis.* By NOEL DEAN BARDSWELL, M.D., and JOHN ELLIS CHAPMAN. 1908. Price, 6s. net.
*Diseases of the Spinal Cord.* By R. T. WILLIAMSON, M.D. 1908. Price, 15s. net.
*Glandular Enlargement.* By ARTHUR EDMUNDS, M.B., M.S., B.Sc. 1908. Price, 7s. 6d. net.
*The Law in General Practice.* By STANLEY B. ATKINSON, M.A., M.B., B.Sc. 1908. Price, 7s. 6d. net.
*A System of Syphilis.* In six vols. Edited by D'ARCY POWER, M.B., and J. KEOGH MURPHY, M.D., M.C. Vol. I. 1908. Price, 42s. net per vol.

From HENRY J. GLAISHER, London:
*Hay-Fever, Hay-Asthma.* By WILLIAM LLOYD. Second Edition. 1908. Price, 4s. 6d. net.

From WILLIAM GREEN & SONS, Edinburgh:
*Arterial Hypertonus, Sclerosis and Blood-Pressure.* By WILLIAM RUSSELL, M.D. 1907.
*Green's Encyclopædia and Dictionary of Medicine and Surgery.* Vol. VII. [1908.] Price, 15s. net.
*An Introduction to Dermatology.* By NORMAN WALKER, M.D. Fourth Edition. 1908. Price, 9s. 6d. net.

From JOHN LANE, London:
*A Short Practice of Aural Surgery.* By J. ARNOLD JONES, M.B., Ch.B. 1908. Price, 5s. net.

From H. K. LEWIS, London:
*Public Health Laboratory Work.* By HENRY R. KENWOOD, M.B., D.P.H. Fourth Edition. 1908. Price, 10s. net.

From LONGMANS, GREEN AND CO., London:
*Quain's Elements of Anatomy.* Eleventh Edition. Vol. I.—*Embryology.* By T. H. BRYCE. 1908. Price, 10s. 6d. net.

From JAMES NISBET & CO., LIMITED, London:
*Injuries of Nerves and their Treatment.* By JAMES SHERREN, F.R.C.S. 1908. Price, 5s. net.

From GEORGE PULMAN & SONS, LIMITED, London:
*The Ophthalmoscope.* Vol. VI. No. 4. April, 1908.

From JOHN WRIGHT & CO., Bristol:
*Supplementary First Aid to Miners.* By WILLIAM B. ARTHUR. 1908. Price, 6d. net.
*Golden Rules of Venereal Disease.* By C. F. MARSHALL, M.D. [1908.] Price, 1s.

